# Carcinoembryonic antigen levels in pancreatic juice are associated with histological subtypes of intraductal papillary mucinous neoplasm of the pancreas

**DOI:** 10.1002/deo2.169

**Published:** 2022-10-11

**Authors:** Hiroshi Hayakawa, Mitsuharu Fukasawa, Shinichi Takano, Hiroko Shindo, Ei Takahashi, Satoshi Kawakami, Yoshimitsu Fukasawa, Natsuhiko Kuratomi, Tadashi Sato, Makoto Kadokura, Sumio Hirose, Shinya Maekawa, Taisuke Inoue, Tatsuya Yamaguchi, Shota Harai, Hiromichi Kawaida, Hiroshi Kono, Kunio Mochizuki, Nobuyuki Enomoto

**Affiliations:** ^1^ First Department of Internal Medicine Faculty of Medicine, Graduate School of Medicine University of Yamanashi Yamanashi Japan; ^2^ First Department of Surgery Faculty of Medicine, Graduate School of Medicine University of Yamanashi Yamanashi Japan; ^3^ Department of Pathology Faculty of Medicine, Graduate School of Medicine University of Yamanashi Yamanashi Japan

**Keywords:** intraductal papillary mucinous neoplasm, histological subtype, endoscopic retrograde pancreatography, carcinoembryonic antigen, pancreatic juice

## Abstract

**Background:**

The present study aimed to examine the correlation between preoperative carcinoembryonic antigen levels in pancreatic juice (PJ‐CEA) and the histological subtype of intraductal papillary mucinous neoplasm (IPMN).

**Methods:**

We enrolled IPMN patients who underwent endoscopic retrograde pancreatography between March 2002 and March 2018. Clinical factors associated with IPMN histological subtypes of 67 patients who underwent surgery were analyzed. Furthermore, the relationship between CEA immunohistochemistry findings and histological subtypes was investigated.

**Results:**

Median PJ‐CEA were 15 ng/ml in the gastric type, 150 ng/ml in the intestinal type, and 175 ng/ml in the pancreatobiliary type. Both intestinal and pancreatobiliary types had significantly higher PJ‐CEA than the gastric type (*p* = 0.001). In the analysis of histological subtype predictors, high PJ‐CEA (≥63 ng/ml) only showed a significant difference in multivariate analyses (95% confidence interval 4.8–70.2; *p* < 0.001). Immunohistochemistry findings revealed significantly higher CEA expression in the non‐gastric type than in the gastric type (*p* < 0.001). The non‐gastric type showed a significantly worse prognosis than the gastric type (*p* = 0.017).

**Conclusion:**

PJ‐CEA was an independent predictor of IPMN histological subtypes in a preoperative setting. High PJ‐CEA predict the non‐gastric type, while low PJ‐CEA predict the gastric type.

## INTRODUCTION

Intraductal papillary mucinous neoplasm (IPMN) presents a wide spectrum of atypia ranging from low‐grade dysplasia (LGD) to invasive carcinoma (IC). According to international consensus guidelines revised in 2017, imaging study findings regarding the presence of a mural nodule, the diameter of the main pancreatic duct, and pancreatic duct involvement account for most clinical factors used to discriminate malignant from benign lesions.^1^ However, even in cases with high‐risk stigmata (an indication for surgery), the malignancy rate is not high, so there is a limit to the preoperative diagnosis potential by imaging alone.^2^

Recently, the histological classification of IPMN has been proposed and reported to reflect differences in malignant potential and prognosis.^3^, ^4^ The gastric type has a good prognosis, but types other than gastric (intestinal and pancreatobiliary) have a high malignancy rate and poor prognosis. IPMN histologic subtype is determined using pathological tissue after surgery. If the histological subtype could be predicted preoperatively, it would be useful to decide whether pancreatic surgery should be performed.

Several studies have reported that carcinoembryonic antigen levels in pancreatic juice (PJ‐CEA) are related to the IPMN histological grade. We previously reported that determining PJ‐CEA is not only useful for malignancy diagnosis but also for the prediction of IPMN malignant transformation without malignant findings on imaging.^5^, ^6^, ^7^, ^8^ However, the relationship between PJ‐CEA and IPMN subtypes has not been sufficiently assessed. This study was designed to elucidate the correlation between preoperative PJ‐CEA and IPMN histological subtype.

### Patients

The present study was conducted with the approval of the ethical review board of the University of Yamanashi. This retrospective observational study enrolled 136 IPMN patients who underwent endoscopic retrograde pancreatography for examining the pancreatic duct and PJ‐CEA between March 2002 and March 2018.

**FIGURE 1:**
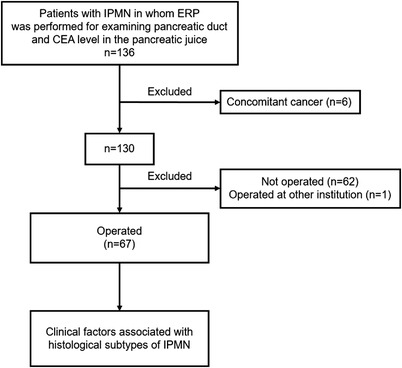
Flowchart of this study. IPMN, intraductal papillary mucinous neoplasm; ERP, endoscopic retrograde pancreatography

A flow chart of the study is presented in Figure 1. We excluded six patients with concomitant carcinoma.

We examined the clinical factors associated with histological subtypes of IPMN in 67 patients who underwent surgery.

The branch duct (BD) type was defined as BD dilatation (≥5 mm) communicating with the main pancreatic duct without main pancreatic dilatation (<5 mm); the main duct (MD) type, as main pancreatic duct dilatation (≥5 mm) without BD dilatation (<5 mm); and the mixed type, as main pancreatic duct dilatation (≥5 mm) with BD dilatation (≥5 mm) communicating with the main pancreatic duct.

Endoscopic retrograde pancreatography was performed in patients with worrisome features or those who required the evaluation of MD involvement using pancreatography or intraductal ultrasonography to determine the surgical excision line.

Surgery is indicated for the following criteria according to international guidelines: obstructive jaundice, the diameter of the main pancreatic duct ≥10 mm, presence of contrast‐enhanced mural nodules measuring ≥5 mm, presence of MD involvement, positive pancreatic juice cytology, and recurrent mucinous pancreatitis.

## METHODS

We evaluated the following in 67 patients who underwent surgery^1^: clinical factors associated with IPMN histological subtypes and^2^ CEA immunohistochemistry staining (IHC) findings of the histological subtypes.

All patients underwent clinical evaluation. Laboratory testing includes serum tumor marker levels, computed tomography (CT), magnetic resonance cholangiopancreatography, and endoscopic ultrasonography (EUS). The size of the branch cyst and the main pancreatic duct diameter were measured using CT or magnetic resonance cholangiopancreatography, whereas mural nodules were assessed using EUS.

Pancreatic juice cytology and CEA concentration measurements were performed using pancreatic juice collected from the main pancreatic duct during preoperative endoscopic retrograde pancreatography. Methods for obtaining pancreatic juice and CEA measurements were reported previously.^8^

Pancreatic juice cytology was classified as classes I–V according to the degree of structural and cytological dysplasia, whereas classes IV and V were defined as positive.^9^ The severity of post‐endoscopic retrograde cholangiopancreatography pancreatitis (PEP) was defined using Cotton's criteria.^10^

Information pertaining to the patient background, imaging findings, and pathological findings was collected from the electronic medical record system. Resected specimens were cut into 5 mm slices after fixation with a formaldehyde solution. The slices were embedded in paraffin and cut into 3 μm sections for hematoxylin and eosin and IHC staining. MUC1 (dilution 1:50, Novocastra; Leica, Nussloch, Germany), MUC2 (dilution 1:100, Novocastra; Leica), MUC5AC (dilution 1:50, Novocastra; Leica), CEA (dilution 1:25, CloneⅡ‐7; Dako, Santa Clara, CA, USA), and Ki‐67 (dilution 1:75, Clone MIB‐1; Dako) were used for IHC. CEA expression in IHC was scored as follows: negative (<50% positive cells) and positive (≥50% positive cells).

Pathological diagnosis was performed in accordance with 2019 WHO classification, which defined LGD as benign and high‐grade dysplasia (HGD) and IC as malignant.^11^

Histological subtypes were classified into gastric, intestinal, and pancreatobiliary types according to WHO classification.^12^

### Statistical analysis

Receiver operator characteristic curve analysis was used to determine the cut‐off value for PJ‐CEA to predict histological subtypes. Univariate analysis of IHC positivity and factors associated with histological subtypes were performed using a Chi‐squared test. A multivariate analysis was performed by using a logistic regression model. Multivariate analysis was performed for the studies with *p*‐values below 0.2 in the univariate analysis. Disease‐specific survival rates were estimated using the Kaplan‐Meier method, and a comparison between the two groups was performed using the log‐rank test. All statistical analyses were performed with the BellCurve for Excel software (version 2.20; Social Survey Research Information Co., Ltd., Tokyo, Japan). Statistical significance was set at *p* < 0.05.

## RESULTS

### Characteristics of the patients

The characteristics of 67 patients are presented in Table 1. Their median (range) age was 69 (37–83) years, and 45 patients (67%) were male. The main IPMN lesion was in the pancreatic head, seen in 33 patients (49%). The IPMN type distribution was as follows: BD type in 20 patients, MD type in 10 patients, and mixed type in 37 patients. The pathological diagnosis was LGD in 33 patients (49%), HGD in 18 patients (27%), and IC in 16 patients (24%). Histological subtypes were gastric type in 42 patients (63%), intestinal type in 17 patients (25%), and pancreatobiliary type in eight patients (12%). The median size (range) of branch cysts was 27 (0–139) mm, the median diameter of the main pancreatic duct (range) was 6 (1.2–33) mm, the median mural nodule size (range) was 5.5 (0–25) mm, and the median PJ‐CEA (range) was 29 (1.2–25,991) ng/ml. Pancreatic juice cytology was positive in nine patients (14%). Seven patients (10.4%) developed PEP. Severity was mild in six patients, and moderate in one patient. There was no severe PEP. All cases improved with conservative treatment.

**TABLE 1:** Patient's characteristics

Characteristics	Resected IPMN No. (%) (*n* = 67)
Age (years), median (range)	69 (37–83)
Sex, male	45 (67)
Location of the main lesion (Ph:Pbt)	33:34
Serum tumor marker	
Elevated CEA	14 (21)
Elevated CA19‐9	8 (12)
Pathological diagnosis	
Low‐grade dysplasia	33 (49)
High‐grade dysplasia	18 (27)
Invasive IPMN	16 (24)
Subtype	
Gastric	42 (63)
Intestinal	17 (25)
Pancreatobillialy	8 (12)
Morphological type	
Branch duct	20 (30)
Main duct	10 (15)
Mixed	37 (55)
Size of branch cyst, median (range), mm	27 (0–139)
Diameter of the main pancreatic duct, median (range), mm	6 (1.2–33)
Size of the mural nodule, median (range), mm	5 (0–25)
CEA levels in the pancreatic juice, median (range), ng/ml	29 (1–25,991)
Pancreatic juice cytology, Class IV/V	9 (14)^a^

Abbreviations: CEA, carcinoembryonic antigen; CA19‐9, carbohydrate antigen; IPMN, intraductal papillary mucinous neoplasm.

^a^ Of 66 patients.

### Relationship between PJ‐CEA and IPMN histological subtypes

**FIGURE 2:**
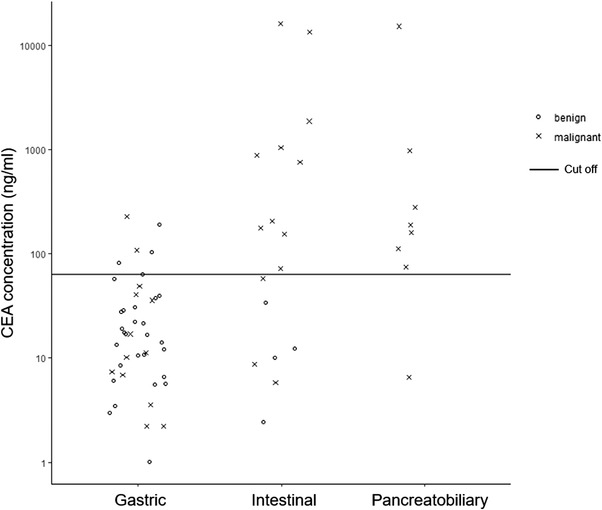
Carcinoembryonic antigen (CEA) concentration in pancreatic juice (log scale) according to pathological subtypes of intraductal papillary mucinous neoplasms. Black line shows the cut‐off value. Cross points show patients with malignant. Round points show the patients with benign

PJ‐CEA according to histological subtypes is presented in Figure 2. The pathological diagnosis of gastric type was LGD in 29 patients (69%), HGD in nine patients (21%), and IC in four patients (10%). The pathological diagnosis of intestinal type was LGD in four patients (24%), HGD in eight patients (47%), and IC in five patients (29%). The pathological diagnosis of the pancreatobiliary type was LGD in zero patients, HGD in one patient (12%), and IC in seven patients (88%). Median PJ‐CEA (range) was 15 ng/ml (1.2–227) in the gastric type, 150 ng/ml (4.5–25,991) in the intestinal type, and 175 ng/ml (5.3–17,953) in the pancreatobiliary type. Both intestinal and pancreatobiliary types had significantly higher PJ‐CEA than the gastric type (*p* = 0.001). PJ‐CEA for diagnosing histological subtypes (gastric or non‐gastric types) was set at 63 ng/ml with the receiver operator characteristic curve (Figure S1). Figure S2

Univariate and multivariate analyses of histological subtype predictors are presented in Table 2. To verify the histological subtype predictor, candidate risk factors were age, gender, IPMN lesion, size of branch cyst, the diameter of the main pancreatic duct, mural nodule size, PJ‐CEA, pancreatic juice cytology, and histological grade. Multivariate analysis showed that high PJ‐CEA (≥63 ng/ml) (odds ratio [OR] 11.5; 95% confidence interval [CI] 2.8–47.1; *p* < 0.001) and histological grade (OR 5.7; 95% CI 1.4–24; *p* < 0.001) were a significant predictor of non‐gastric type IPMN.

**TABLE 2:** Factors associated with intraductal papillary mucinous neoplasm subtype

		**IPMN subtype**				
		**Non‐gastric**	**Gastric**	**Univariate analysis**	**Multivariate analysis**	
**Characteristics**		**(*n* = 25)**	**(*n* = 42)**	***p*‐value**	***p*‐value**	**Odds ratio (95% CI)**
Age, year	≧75	11	10	0.11	0.25	2.27 (0.6–9.3)
	<75	14	32			
Sex	Male	16	29	0.79		
	Female	9	13			
Location of tumor	Ph	12	21	1		
	Pb/Pt	13	21			
Size of branch cyst, mm	≧30	11	20	0.81		
	<30	14	22			
Size of mural nodule, mm	≧5	14	20	0.62		
	<5	11	22			
Diameter of main pancreatic duct size, mm	≧5	20	26	0.17	0.34	2.2 (0.4–11.2)
	<5	5	16			
CEA level in the pancreatic juice, ng/ml	≧63	18	6	<0.001	<0.001	11.5 (2.8–47.1)
	<63	7	36			
Pancreatic juice cytology	Positive	4	6	1		
	Negative	21	36			
Pathological diagnosis	LGD	4	29	<0.001	0.02	5.7 (1.4–24)
	HGD/IC	21	13			

Abbreviations: CEA, carcinoembryonic antigen; HGD, high‐grade dysplasia; IC, invasive carcinoma; IPMN, intraductal papillary mucinous neoplasm; LGD, low‐grade dysplasia.

### Correlation between CEA IHC staining and histological subtype

Of the 67 patients, 66 were examined for correlation between CEA expression in IHC and histological subtype (Table 3). In the gastric type, CEA expression was positive in six patients (14%) and negative in 36 patients (86%). In the non‐gastric type, CEA expression was positive in 20 patients (83%) and negative in four patients (17%). CEA expression in the non‐gastric type was statistically higher than in the gastric type (*p* < 0.001).

**TABLE 3:** Association of a histologic subtype with carcinoembryonic antigen (CEA)

**IHC**	**Expression**	**Gastric**	**Non‐gastric**	***p*‐value**
CEA	Negative (–)	36	4	<0.001
	Positive (+)	6	20	

Abbreviation: CEA, carcinoembryonic antigen.

### Cases with gastric and non‐gastric type

**FIGURE 3:**
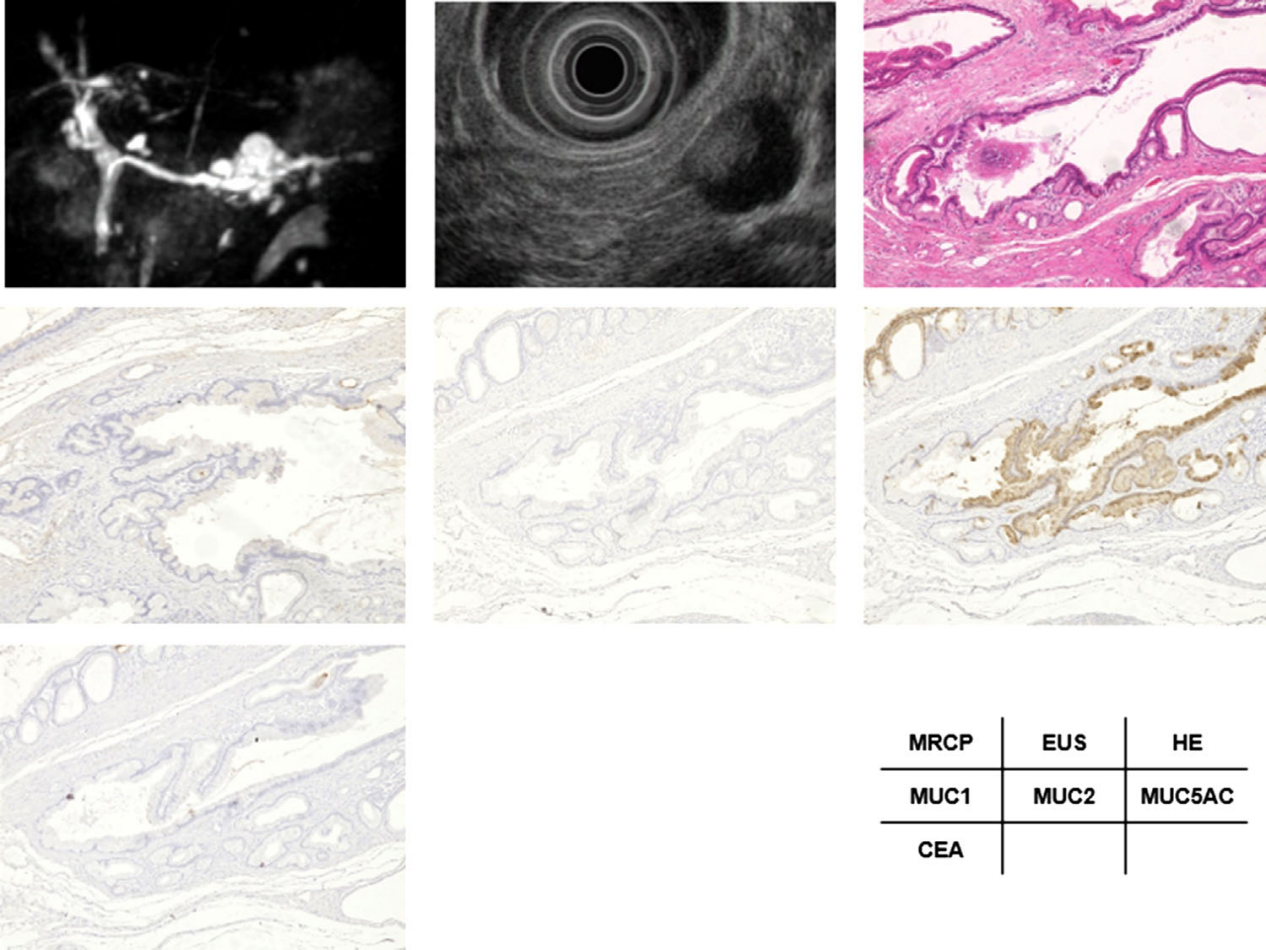
A 79‐year‐old male with mixed‐intraductal papillary mucinous neoplasm (IPMN) and low pancreatic juice carcinoembryonic antigen (CEA) levels (15 ng/ml). (a) The diameter of the main pancreatic duct was 6 mm. (b) Endoscopic ultrasonography (EUS) showed a mural nodule 8 mm in height in the branch duct. (c) H&E staining (4 ×) showed papillary proliferation of moderate atypical cells found in the dilated branch duct. (d) MUC1 staining (4 ×) was negative. (e) MUC2 staining (4 ×) was negative. (f) MUC5AC staining (4 ×) was diffusely positive. (g) CEA staining (4 ×) was negative. The pathological diagnosis of the resected tissue was low‐grade dysplasia (gastric type).

A case with gastric type IPMN is presented in Figure 3. A 79‐year‐old male displayed mixed‐IPMN with low pancreatic juice CEA levels (15 ng/ml). The diameter of the main pancreatic duct was 6 mm. EUS showed a mural nodule with 8 mm in height in the BD. Hematoxylin and eosin (H&E) staining showed papillary proliferation of moderate atypical cells found in the dilated BD. MUC1 staining was negative, MUC2 staining was negative, MUC5AC staining was diffusely positive, and CEA staining was negative. The pathological diagnosis of the resected tissue was LGD (gastric type).

**FIGURE 4:**
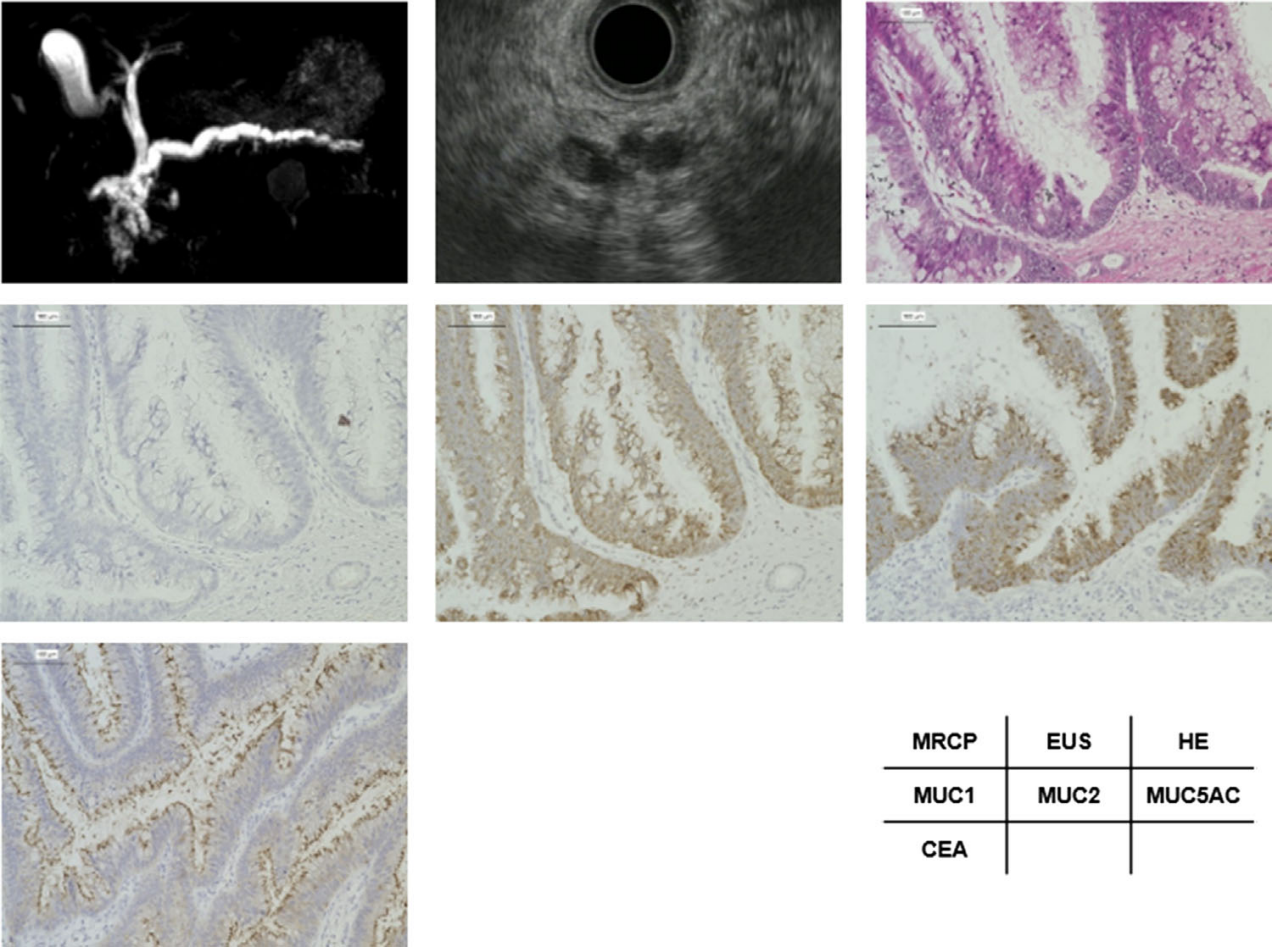
A 66‐year‐old male with high pancreatic juice carcinoembryonic antigen (CEA) levels (102 ng/ml) had MD‐IPMN around the pancreas head. (a, magnetic resonance cholangiopancreatography) The diameter of the main pancreatic duct was 8 mm. (b) Endoscopic ultrasonography (EUS) showed a mural nodule 4 mm in height in the main pancreatic duct. (c) Hematoxylin and eosin (H&E) staining (10 ×) showed papillary proliferation of atypical cells found in the dilated main pancreatic duct. (d) MUC1 staining (10 ×) was negative. (e) MUC2 staining (10 ×) was diffusely positive. (f) MUC5AC staining (10 ×) was diffusely positive. (g) CEA staining (10 ×) was positive on the luminal surface of the neoplastic epithelium. The pathological diagnosis of the resected tissue was high‐grade dysplasia (intestinal type)

A case with non‐gastric type IPMN is presented in Figure 4. A 66‐year‐old male displayed MD‐IPMN with high pancreatic juice CEA levels (102 ng/ml). The diameter of the main pancreatic duct was 8 mm. EUS showed a mural nodule with 4 mm in height in the main pancreatic duct. H&E staining showed papillary proliferation of atypical cells found in the dilated main pancreatic duct. MUC1 staining was negative, MUC2 staining was diffusely positive; MUC5AC staining was diffusely positive, and CEA staining was positive on the luminal surface of the neoplastic epithelium. The pathological diagnosis of the resected tissue was HGD (intestinal type).

### Survival of patients with gastric type and non‐gastric type

Disease‐specific survival rates were estimated using the Kaplan‐Meier method. The follow‐up period was started from surgery. The median follow‐up period was 47 months. Of the 67 patients, nine patients developed recurrences. Among them, eight patients died from distant metastasis. One patient who developed recurrence showed IPMN in the remnant pancreas and underwent a total pancreatectomy survived. Of the eight patients who died, two patients had the gastric type, one patient had the intestinal type, and 5 patients had the pancreatobiliary type. The pathological diagnosis of all patients who died was IC. Patients with the gastric type had a fair prognosis (5‐year survival rates of 0.929 (95% CI 0.83–1.00)). Patients with the non‐gastric type had relatively poor prognoses (5‐year survival rates of 0.776 (95% CI 0.6–0.95)) (Figure 2). Patients with the non‐gastric type showed a significantly worse prognosis than those with the gastric type (*p* = 0.017).

## DISCUSSION

In the present study, PJ‐CEA in the non‐gastric type was significantly higher than that in the gastric type. In addition, PJ‐CEA were a preoperative predictor of IPMN histological subtypes. In the IHC analysis, CEA expression in the non‐gastric type was significantly higher than in the gastric type. In the prognosis analysis, disease‐specific survival rates in patients with the non‐gastric type were lower than those in patients with the gastric type.

In the previous reports of IPMN histological subtypes, subtypes were associated with histological grade and prognosis. The gastric type was associated with the involvement of the BD, a lower historical grade, the absence of invasion, and fair survival. The intestinal type was associated with involvement of the MD, HGD, and a less favorable prognosis. The pancreatobiliary type was associated with a high historical grade, invasive tubular adenocarcinoma, and a poor prognosis.^4^, ^13^, ^14^, ^15^ In the current study, the prognosis in the non‐gastric type was worse than in the gastric type. This result was consistent with those reported by previous studies.

To the best of our knowledge, there are no reports on the association between PJ‐CEA and histological IPMN subtypes. In a report examining CEA concentration in cyst fluid obtained from EUS‐guided fine‐needle aspiration, there was a significant correlation between CEA concentration in cyst fluid and CEA IHC findings.^16^ It was reported that intestinal type was associated with high cyst fluid CEA. Meanwhile, another report suggested that gastric type is associated with significantly high cyst fluid CEA.^17^

There is no consensus about the association between cyst fluid CEA and histological subtypes.

In the present study, low PJ‐CEA were associated with the gastric type and high PJ‐CEA were associated with the non‐gastric type. This result is consistent with the IHC findings and can be considered reliable. These results suggest that examining PJ‐CEA is an effective option to predict histological subtypes.

In a previous report on the preoperative diagnosis of histological BD‐IPMN subtypes, pancreatic juice cytology using the cell block method and immunostaining was found to be a useful diagnostic method.^18^ However, histological subtype diagnosis based on pancreatic juice cytology needs to be evaluated by pathologists with specific expertise in pancreatic pathology. However, in some institutions without expert pathologists, it may be difficult to diagnose histological subtypes. PJ‐CEA has an obvious cut‐off value, moreover, the method for PJ‐CEA is the same as that for measuring CEA in serum. As a result, PJ‐CEA can be widely used across many institutions.

When collecting pancreatic juice, PEP is the most worrisome adverse event. In the present study, some patients developed PEP. Most PEP severity was mild and there were no severe cases.

We previously reported that PJ‐CEA were associated with a future IPMN malignant transformation. In the present study, high PJ‐CEA were associated with the non‐gastric type of IPMN. Therefore, it is speculated that non‐gastric cases with a malignant potential, which are often found in the high PJ‐CEA group, are associated with a future malignant transformation.

The present study had several limitations. First, this was a single‐center, retrospective observational study with a small sample size. Second, a selection bias may have occurred because of the selection of cases that underwent surgery. Third, it has been reported that some IPMNs have overlapping subtypes.^19^, ^20^ It may be difficult to determine the subtype using PJ‐CEA in those cases. However, measuring preoperative PJ‐CEA and predicting histological subtypes may be useful in predicting high‐risk cases, surveillance, and surgical decisioning.

In conclusion, PJ‐CEA were an independent predictor of predicting histological subtypes of IPMN in preoperative. Although larger studies are necessary to confirm these results, high PJ‐CEA predict the non‐gastric type, and low PJ‐CEA predict the gastric type.

## CONFLICT OF INTEREST

The authors declare that they have no conflict of interest.

## FUNDING INFORMATION

None.

## Supporting information


**Supplementary Figure 1**: The receiver operating characteristic curve used to determine the CEA cut‐off level in the pancreatic juice to predict histological subtypes. The area under the curve level was 0.8. The cut‐off level was set at 63 ng/ml.

**Supplementary Figure 2**: Kaplan‐Meier survival curves to show the cumulative survival of patients with intraductal papillary mucinous neoplasms according to pathological subtypes (gastric vs non‐gastric; p = 0.02, log‐rank test).
