# Supplementary figures and images for: Carcinoembryonic antigen levels in pancreatic juice are associated with histological subtypes of intraductal papillary mucinous neoplasm of the pancreas
Source: DEN Open. 2022 Oct 11;3(1):e169. doi: 10.1002/deo2.169 (PMC9552336; doi:10.1002/deo2.169)

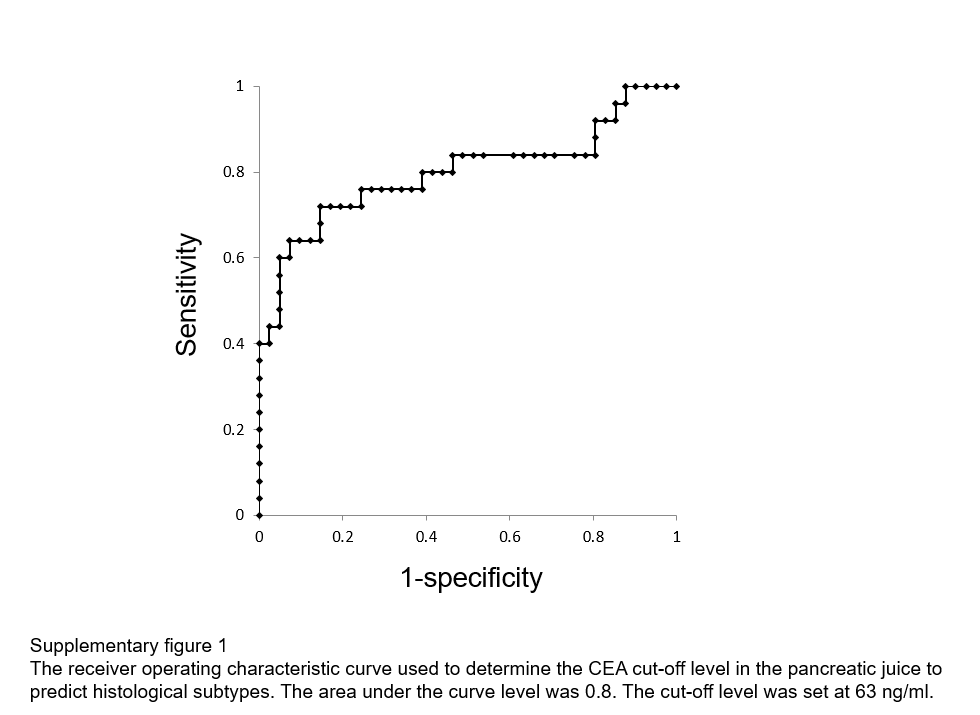

Supplement: Supplementary file 1 — Supplementary Figure 1: The receiver operating characteristic curve used to determine the CEA cut‐off level in the pancreatic juice to predict histological subtypes. The area under the curve level was 0.8. The cut‐off level was set at 63 ng/ml. [file DEO2-3-e169-s002.tif]

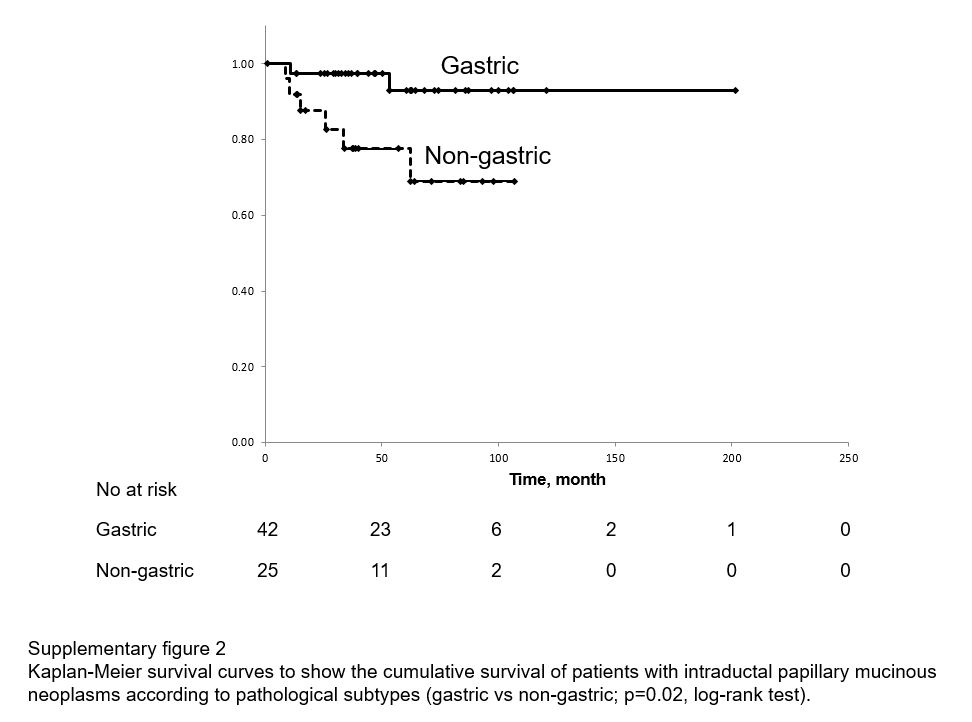

Supplement: Supplementary file 2 — Supplementary Figure 2: Kaplan‐Meier survival curves to show the cumulative survival of patients with intraductal papillary mucinous neoplasms according to pathological subtypes (gastric vs non‐gastric; p = 0.02, log‐rank test). [file DEO2-3-e169-s001.tif]
